# High MCM8 expression correlates with unfavorable prognosis and induces immune cell infiltration in hepatocellular carcinoma

**DOI:** 10.18632/aging.204440

**Published:** 2022-12-27

**Authors:** Meng Yu, Huaxiang Wang, Hongyang Xu, Yuhang Lv, Qingsong Li

**Affiliations:** 1 Department of Critical Care Medicine, Taizhou Central Hospital (Taizhou University Hospital), Taizhou 318000, Zhejiang, China; 2 Department of Hepatobiliary and Pancreatic Surgery, Taihe Hospital, Affiliated Hospital of Hubei University of Medicine, Shiyan 442000, Hubei, China; 3 Department of Gastroenterology, Taizhou Central Hospital (Taizhou University Hospital), Taizhou 318000, Zhejiang, China

**Keywords:** MCM8, hepatocellular carcinoma, genetic alteration, DNA methylation, immune infiltrates

## Abstract

Background: MCM8 has been reported highly expressed in several human malignancies. However, its role in HCC has not yet been researched.

Methods: The prognostic significance of MCM8 mRNA expression was analyzed using datasets from TCGA and GEO databases. Immunohistochemistry (IHC) assay was used to detect the MCM8 protein expression in HCC tissues. The Cox regression analysis was employed to determine the independent prognostic value of MCM8. Then, we established a nomogram for OS and RFS prediction based on MCM8 protein expression. We analyzed the DNA methylation and genetic alteration of MCM8 in HCC. Moreover, GO, KEGG and GSEA were utilized to explore the potential biological functions of MCM8. Subsequently, we evaluate the correlations between MCM8 expression and composition of the tumor microenvironment as well as immunocyte infiltration ratio in HCC.

Results: MCM8 mRNA and protein were significantly overexpressed in HCC tissues. High MCM8 protein expression was an independent risk factor for OS and RFS of HCC patients. MCM8 expression is altered in 60% of queried HCC patients. In addition, higher methylation of the CpG site cg03098629, cg10518808, and 17230679 correlated with lower MCM8 levels. MCM8 expression correlated with cell cycle and DNA replication signaling. Moreover, MCM8 may be correlated with different compositions of the tumor microenvironment and immunocyte infiltration ratio in HCC.

Conclusions: MCM8 was highly expressed in HCC tissues and was associated with poor prognosis. Meanwhile, high expression of MCM8 may induce immune cell infiltration and may be a promising prognostic biomarker for HCC.

## INTRODUCTION

Hepatocellular carcinoma was one of the most common and deadliest malignancies in the digestive system, which causes an immense economic and health burden worldwide, especially in South Asia [1, 2]. Despite advances in immunotherapy and targeted molecular have improved the treatment of HCC in the recent decade, the 5-year overall survival rate was still less than 20% [3, 4]. The low survival rate in patients with HCC was specifically due to the low detection rate in the early stages of the disease and the early recurrence after curative resection [5]. Although biomarkers with diagnostic and prognostic values have been continuously identified in recent years, their sensitivity and specificity are the main limitations for clinical application [6, 7]. Therefore, it is imperative to identify new diagnostic and prognostic molecular biomarkers and uncover their molecular mechanisms and signaling pathways to improve the prognosis of patients with HCC.

DNA replication has been shown to control the tumorigenesis and proliferation of cancers through multiple mechanisms and was considered to be one of the hallmarks acquired by cancer cells [8, 9]. Mini-chromosome Maintenance family (MCMs) are core proteins that constitute the DNA replication licensing complex and play a key regulatory role in the replication of each cell cycle [10, 11]. MCM6 was identified to be a promoter that drives S/G2 cell cycle progression and was associated with poor survival in HCC patients [12]. In addition, MCM2-7 was revealed to be involved in the proliferation of HCC cells and plays an important role in pathogenesis [13]. Moreover, High expression of MCM3 enhanced radioresistance via activating the NF-κB pathway thereby facilitating invasion and metastasis of HCC [14]. Previous studies proposed that MCM4 and MCM10 also act as potential biomarkers and promote cell proliferation in HCC [15, 16]. MCM8, an important helicase involved in the elongation step of DNA replication, was associated with chromosomal instability [17–19]. Several studies have indicated that the mutation in the MCM8 gene was involved in primary ovarian insufficiency and short stature [20–22]. MCM8 have been found to be aberrantly expressed in a variety of malignancies including gastric cancer [23], cholangiocarcinoma [24], glioblastomas [25], bladder cancer [26], osteosarcoma [27] and myeloid tumors [28]. However, rarely study comprehensively investigated the role and the mechanism of action of MCM8 in the tumorigenesis and progression of HCC.

In this study, we systematically investigated the clinical prognostic significance and function of MCM8. We analyzed the mRNA expression of MCM8 and its association with clinical prognosis using datasets from The Cancer Genome Atlas (TCGA) and the Gene Expression Omnibus (GEO). Immunohistochemistry (IHC) assay was employed to validate the protein expression in HCC tissue. The Cox regression analysis was used to determine the independent prognostic value of MCM8, and then we constructed a nomogram to predict the overall survival and recurrence-free survival, respectively. We also analyzed the associations of genetic alteration of MCM8 with OS and RFS in patients with HCC. Moreover, we searched for CpG sites and determined the relationship between gene expression with DNA methylation in MCM8 as well as OS in HCC patients. It is widely accepted that one method of inferring gene function is co-expression analysis. In addition, genes with similar expression patterns may be functionally similar. Therefore, we identified the co-expressed genes of MCM8 to further explore the gene functions. The Gene Ontology (GO), Kyoto Encyclopedia of Genes and Genomes (KEGG) and Gene Set Enrichment Analyses (GSEA) were utilized to explore the potential biological functions of MCM8. Finally, we evaluate the correlations between MCM8 expression and composition of the tumor microenvironment as well as immunocyte infiltration ratio in HCC using CIBERSORT and ssGSEA algorithms.

## RESULTS

### MCM8 mRNA expression was significantly elevated and predicted poor prognosis in patients with HCC

**Figure 1:**
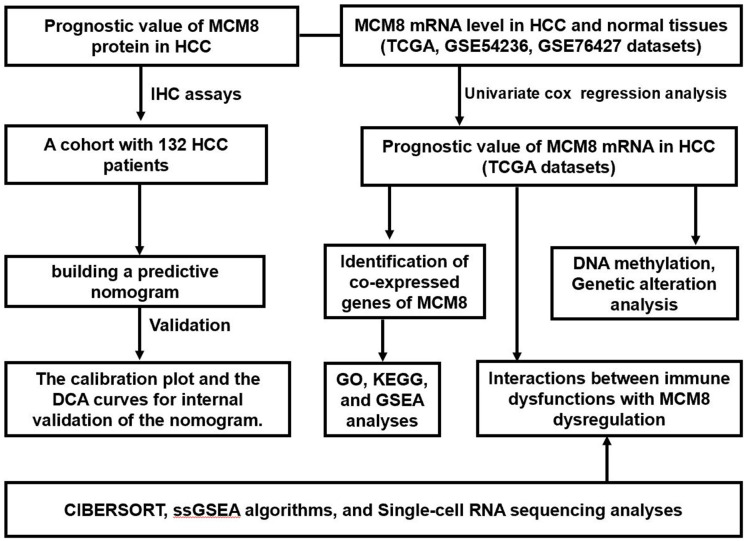
The flow chart presented the overview of the steps in our study.

**Figure 2:**
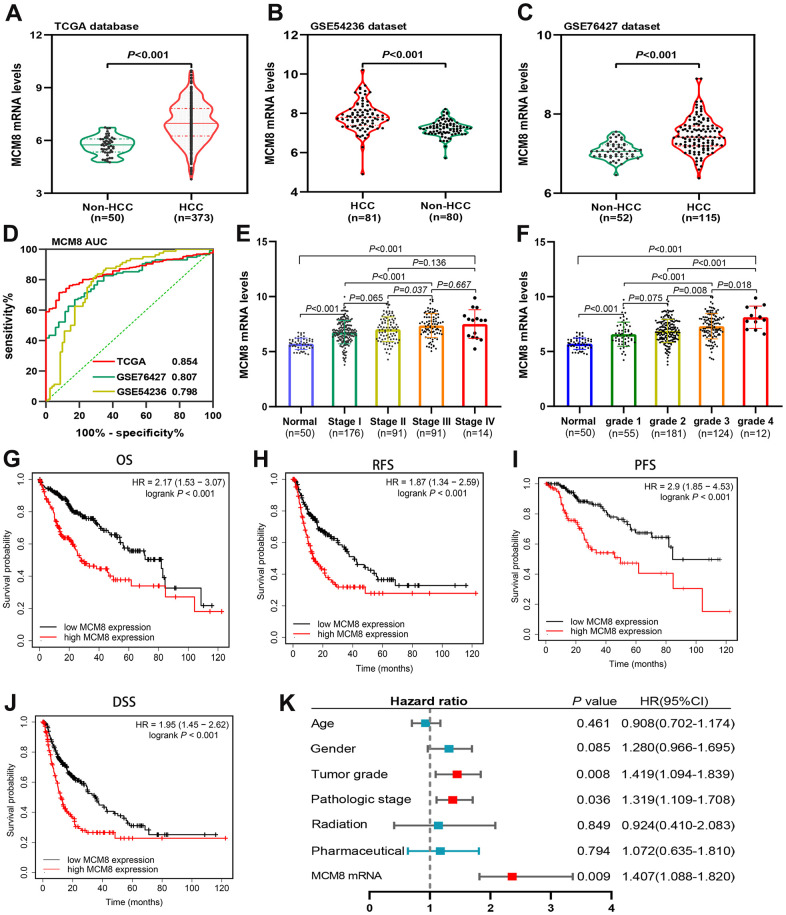
**MCM8 mRNA levels in HCC and adjacent normal liver tissues and its prognostic value.** (**A**–**C**) MCM8 mRNA was up-regulated in HCC tissues in TCGA (**A**), GSE54236 (**B**) and GSE76426 (**C**) datasets. (**D**) ROC curve shows the diagnostic significance of MCM8 mRNA for HCC in TCGA and GEO databases. (**E**, **F**) The MCM8 mRNA levels gradually increased with the progression of tumor stage (**E**) and grade (**F**). (**G**–**J**) High MCM8 expression was correlated with poor OS (**G**), RFS (**H**), PFS (**I**) and DSS (**J**) in HCC. (**K**) Higher MCM8 mRNA level was one of the independent risk factors for OS in TCGA database.

Figure 1 showed the schematic diagram of our study (Figure 1). We investigated the mRNA levels of MCM8 in HCC tissues and adjacent normal liver tissues in TCGA and GEO (GSE76427 and GSE54236 datasets) databases and found that its mRNA expression significantly upregulated in HCC (all P<0.001, Figure 2A–2C). In addition, ROC analysis demonstrated that MCM8 mRNA levels exhibited an excellent capacity to discriminate the HCC and normal liver tissues, with area under the curve (AUC) for TCGA, GSE76427 and GSE54236 being 0.854, 0.807 and 0.798, respectively (all P<0.001, Figure 2D). Moreover, the MCM8 mRNA levels gradually increased with the progression of tumor stages (Figure 2E) and histologic grades (Figure 2F) in TCGA database. According to the Kaplan–Meier survival curves, patients with higher MCM8 mRNA levels have remarkably shorter OS, RFS, PFS, and DSS (all P<0.001, Figure 2G–2J). We also performed the multivariate Cox regression analysis and found that a higher MCM8 mRNA level was one of the independent risk factors for OS (P=0.009, HR((95%CI): 1.407(1.088-1.820)) (Figure 1K).

### MCM8 protein expression was upregulated and correlated with poorer survival and clinical outcomes in a cohort of 132 HCC patients

**Figure 3:**
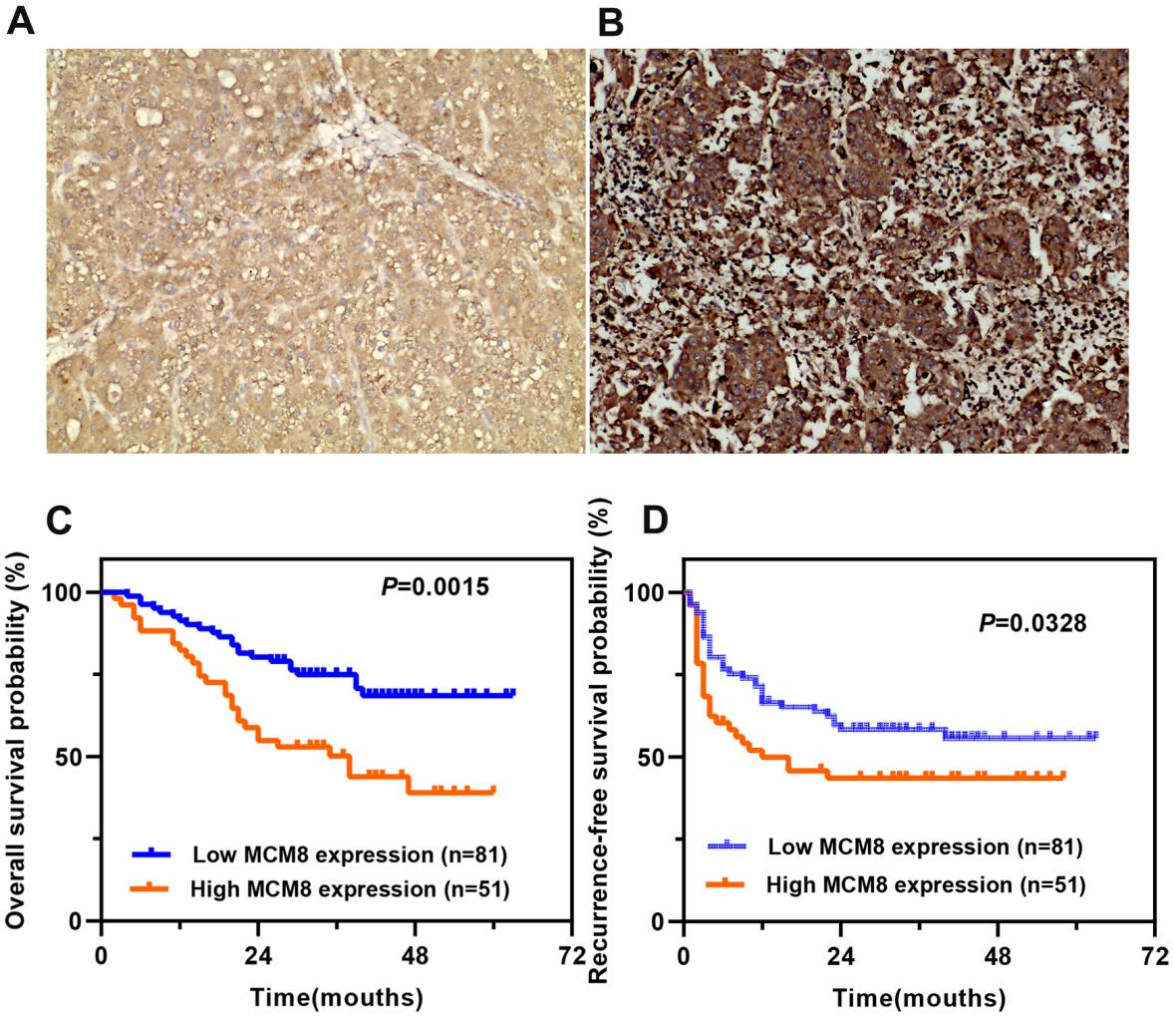
**Prognostic value of MCM8 protein expression in a cohort of 132 HCC patients.** (**A**, **B**) Representative images of low (**A**) and high (**B**) MCM8 protein expression in HCC tissues by IHC staining (×200 magnification). (**C**, **D**) Patients with higher MCM8 protein expression have poorer OS (**C**) and RFS (**D**).

We then investigated the MCM8 protein expression in a cohort of 132 HCC patients and found that MCM8 protein was predominantly localized in the nucleus of the HCC cells. In addition, its expression was remarkably higher in HCC tissues than in the adjacent normal liver tissues. The 132 HCC patients were stratified into high and low MCM8 expression groups (n=81 and 51, respectively) based on their IHC scores. HCC samples with a IHC score of 0, 1 or 2 was defined as low MCM8 expression group, whereas a score of 3 or 4 was defined as high MCM8 expression group. As shown in Figure 3A, 3B, we exhibited the representative IHC images of low and high MCM8 protein expression in HCC tissues (Figure 3A, 3B). We next analyzed the correlation between MCM8 protein expression and clinical outcomes and found that high MCM8 protein level was positively associated with worse TNM staging (P=0.002), vascular invasion (P=0.043), high recurrence rate (P=0.026) and death rate (P=0.002), whereas not associated with age, gender, tumor size, tumor grade, serum AFP level, tumor location, tumor differentiation, etc. (Table 1). The univariate Cox regression analysis demonstrated that greater tumor size (P=0.016), higher TNM stage (P=0.022), Child-Pugh class B (P<0.001), vascular invasion (P=0.006) and higher MCM8 protein level (P=0.002) were risk factors for OS in patients with HCC. For RFS, greater tumor size (P=0.036), lower tumor differentiation (P=0.027), Child-Pugh class B (P=0.014), vascular invasion (P<0.001), without tumor encapsulation (P<0.001) and higher MCM8 protein level (P=0.029) were risk factors (Table 2). Meanwhile, the multivariate Cox regression analysis confirmed that Child-Pugh class B (HR (95%CI) 4.323(2.156-8.668), P <0.001), vascular invasion (HR (95%CI) 2.285(1.226-4.192), P = 0.008) and higher MCM8 protein level (HR (95%CI) 1.816(1.031-3.201), P = 0.039) were independent risk factors for OS in patients with HCC. For RFS, vascular invasion (HR (95%CI) 2.227(1.282-3.869), P = 0.005), without tumor encapsulation (HR (95%CI) 0.246(0.145-0.415), P <0.001) and higher MCM8 protein level (HR (95%CI) 1.657(1.002-2.741), P = 0.049) were independent risk factors (Table 3). Survival curves suggested that patients with higher MCM8 protein levels have shorter OS and RFS probability (Figure 3C, 3D). These results revealed that MCM8 protein expression has an excellent prognostic significance for patients with HCC.

**Table 1:** Correlation between MCM8 protein expression and clinical outcomes in HCC patients(n=132).

**Characteristics**		**N**	**MCM8 level**		**X^2^**	*P-Value
			**High(n)**	**Low(n)**		
Age (year)	>55	85	32	53	0.099	0.754
	<=55	47	19	28		
Gender	Male	114	41	73	2.516	0.113
	Female	18	10	8		
Tumor size (cm)	>5cm	70	26	44	1.400	0.708
	<=5cm	62	25	37		
TNM stage	I/II	72	19	53	10.022	**0.002**
	III	60	32	28		
Tumor grade	G1/G2	26	13	13	1.763	0.184
	G3/G4	106	38	68		
Serum AFP level	>400ng/ml	55	21	34	0.008	0.928
	<=400ng/ml	77	30	47		
Tumor location	Left	86	38	48	3.206	0.073
	Right	46	13	33		
Tumor differentiation	Low	19	8	11	0.496	0.780
	Median	83	33	50		
	High	30	10	20		
Vascular invasion	Yes	69	21	48	4.102	**0.043**
	No	63	30	33		
Tumor encapsulation	Yes	88	34	54	0.000	1.000
	No	44	17	27		
HBV DNA load	>10^4^	59	26	33	1.327	0.249
	<=10^4^	73	25	48		
Child-Pugh class	A	64	22	42	0.952	0.329
	B	68	29	39		
Recurrence	Yes	62	29	30	4.976	**0.026**
	No	70	22	51		
Status	Alive	81	23	58	9.275	**0.002**
	Dead	51	28	23		

TNM, tumor, node, metastasis; AFP, alpha fetoprotein. *P-Value<0.05 were considered statistically significant.

**Table 2:** Univariate Cox regression analysis of overall survival and recurrence-free survival in 132 patients with hepatocellular carcinoma.

**Variables**		**Overall survival**	*P-Value	**Recurrence-free survival**	*P-Value
		**HR(95%CI)**		**HR(95%CI)**	
Age (year)	>55 vs. <=55	1.171(0.667-2.057)	0.583	1.286(0.778-2.128)	0.327
Gender	Male vs. female	1.024(0.461-2.274)	0.954	1.260(0.642-2.475)	0.502
Tumor size (cm)	>5 vs. <=5	2.073(1.144-3.758)	**0.016**	1.723(1.035-2.869)	**0.036**
TNM stage	I/II vs. III	1.899(1.096-3.289)	**0.022**	1.098(0.658-1.830)	0.721
Serum AFP level	>400 vs <=400	1.582(0.913-2.739)	0.102	1.499(0.917-2.450)	0.106
Tumor location	Left vs. right	0.700(0.401-1.223)	0.211	1.308(0.765-2.237)	0.327
Tumor differentiation	High vs. median/low	1.577(0.789-3.150)	0.197	1.997(1.083-3.682)	**0.027**
HBsAg	Positive vs. negative	1.134(0.654-1.966)	0.654	0.907(0.553-1.487)	0.699
Tumor grade	G1/G2 vs. G3/G4	0.861(0.419-1.770)	0.684	0.813(0.425-1.557)	0.532
Child-Pugh class	A vs. B	5.447(2.827-10.493)	**<0.001**	1.865(1.134-3.068)	**0.014**
Vascular invasion	Yes vs. no	2.228(1.254-3.961)	**0.006**	2.512(1.501-0.415)	**<0.001**
Tumor encapsulation	Yes vs. no	0.747(0.425-1.313)	0.311	0.251(0.151-0.415)	**<0.001**
MCM8 protein level	High vs. low	2.370(1.364-4.120)	**0.002**	1.728(1.057-2.827)	**0.029**

HR, Hazard ratio; CI, confidential interval; TNM, tumor, node, metastasis; AFP, alpha fetoprotein. *P-Value<0.05 were considered statistically significant.

**Table 3:** Multivariate Cox regression analysis of overall survival and recurrence-free survival in 132 patients with hepatocellular carcinoma.

**Variables**		**Overall survival**	*P-Value	**Recurrence-free survival**	*P-Value
		**aHR(95%CI)**		**aHR(95%CI)**	
Tumor size (cm)	>5 vs. <=5	1.173(0.579-2.375)	0.665	1.463(0.859-2.494)	0.162
TNM stage	I/II vs. III	1.232(0.623-2.433)	0.549		
Tumor differentiation	High vs. median/low			1.451(0.760-2.771)	0.259
Child-Pugh class	A vs. B	4.323(2.156-8.668)	**<0.001**	1.380(0.814-2.338)	0.232
Vascular invasion	Yes vs. no	2.285(1.226-4.192)	**0.008**	2.227(1.282-3.869)	**0.005**
Tumor encapsulation	Yes vs. no			0.246(0.145-0.415)	**<0.001**
MCM8 protein level	High vs. low	1.816(1.031-3.201)	**0.039**	1.657(1.002-2.741)	**0.049**

aHR, adjusted hazard ratio; CI, confidential interval; TNM, tumor, node, metastasis. *P-Value<0.05 were considered statistically significant.

### Predictive nomogram construction

**Figure 4:**
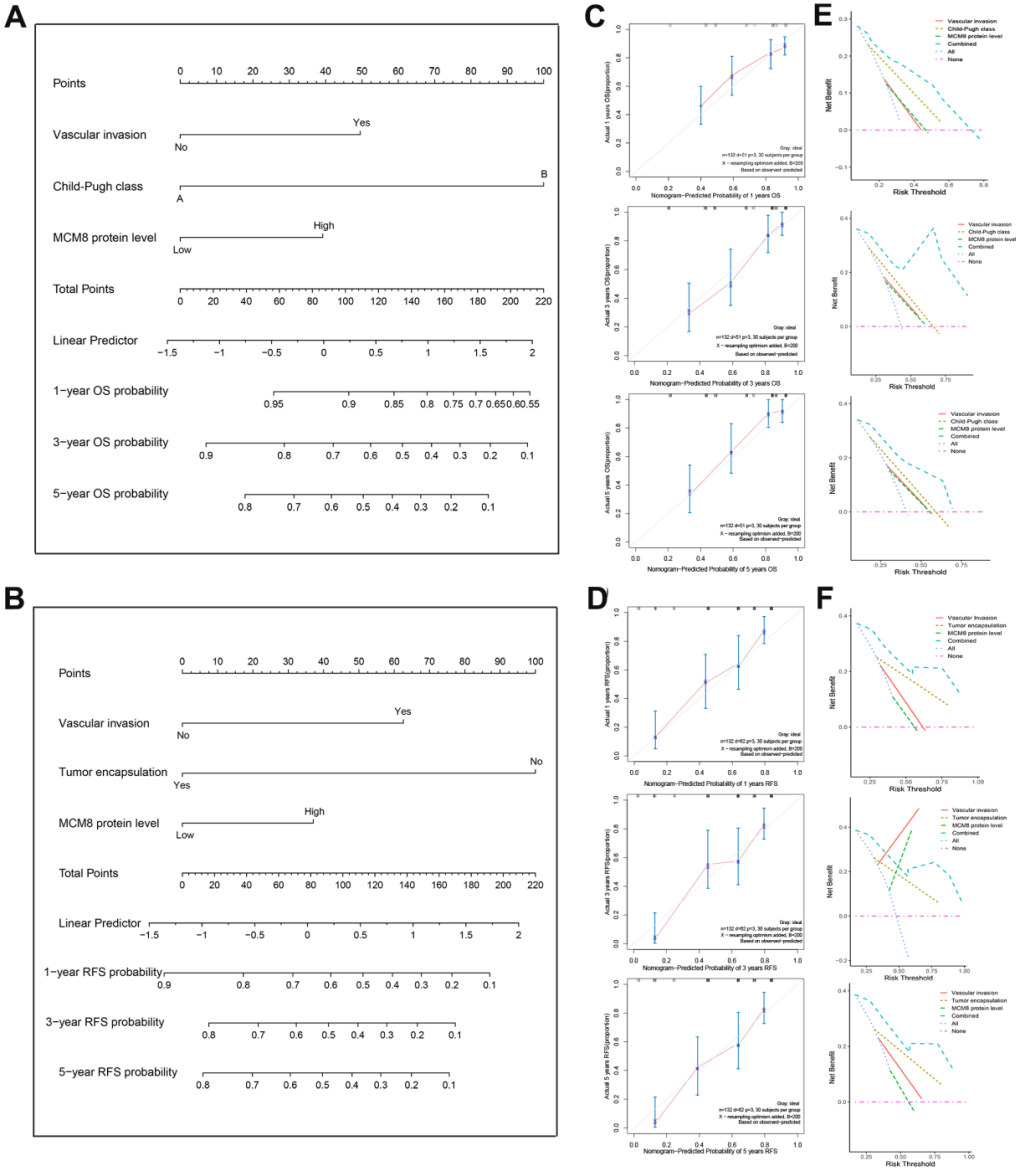
**Construction and validation of the prognostic nomogram.** (**A**, **B**) Nomogram established from a cohort of 132 HCC patients to predict OS (**A**) and RFS (**B**) probability. (**C**, **D**) The calibration plot of the nomogram for predicting the OS (**C**) and RFS (**D**) survival probability at 1-, 3-, and 5-year. (**E**, **F**) DCA curves shown that nomogram exhibited highest net benefit for 1-, 3-, and 5-year OS (**E**) and RFS (**F**) prediction than three single predictive factors.

In order to provide a quantitative approach to accurately predict the OS and RFS probability, two nomograms that integrated the MCM8 protein expression level and other independent risk factors identified by the multivariate Cox regression analysis were established (Figure 4A, 4B). We plotted the calibration curves to evaluate the predicting capacity of the nomogram and showed an excellent prediction performance for 1-, 3-, and 5-year OS and RFS when compared to the ideal model (Figure 4C, 4D). Similarly, using the DCA curves, the nomogram still exhibited a higher net benefit for 1-, 3-, and 5-year OS and RFS prediction than three single predictive factors (Figure 3E, 3F).

### Genetic alterations correlated with dysregulation of MCM8 expression and poorer survival in HCC patients

**Figure 5:**
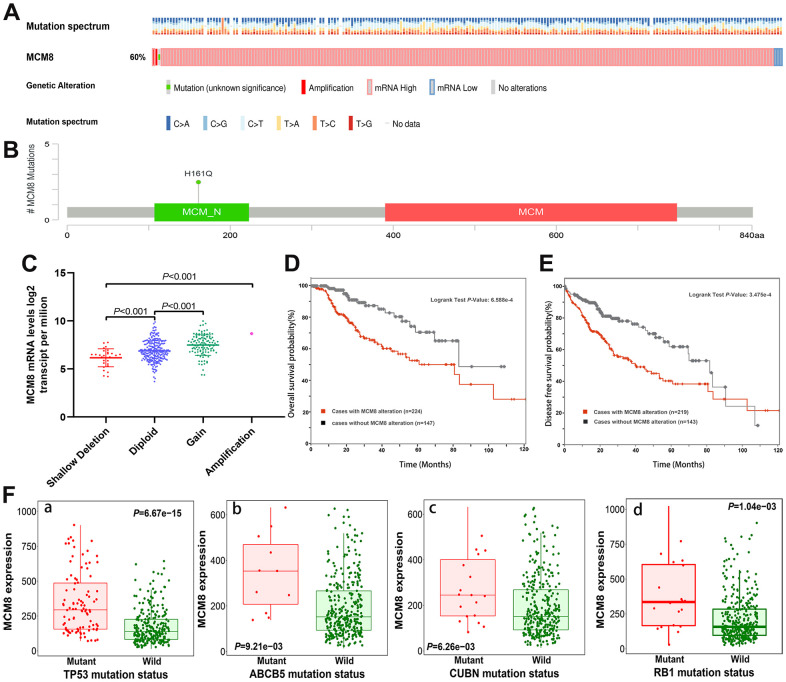
**MCM8 alteration was associated with worse survival in HCC patients.** (**A**) 60% of the queried patients have detected alteration of MCM8 in the cBioPortal database. (**B**) A mutational hotspot of H161Q/Missense was detected in 29 HCC patients. (**C**) Dysregulation of MCM8 mRNA expression correlated with copy number alterations in HCC. (**D**, **E**) Patients with MCM8 alteration have poorer overall survival probability (**D**) and disease-free survival probability (**E**) than patients without genetic alterations. (**F**) Mutation status of TP53 (**a**), ABCB5 (**b**), CUBN (**c**) and RB1 (**d**) were correlated with dysregulation of MCM8 mRNA expression in HCC.

We investigated the genetic alteration of MCM8 in a cohort of 372 HCC patients in the cBioPortal database and found 224 (60%) of the queried patients have detected alteration of MCM8, including 1case of missense mutation, 1 case of amplification, 214 cases of high expression and 8 cases of low expression (Figure 5A). Meanwhile, a mutational hotspot of H161Q/Missense have detected in 29 cases of HCC patients, whereas the somatic mutations of MCM8 were observed in 0.3% of the patients (Figure 5B). In addition, the dysregulation of MCM8 mRNA expression correlated with copy number alterations in the cBioPortal database (Figure 5C). Moreover, the survival analysis revealed that patients with MCM8 alterations have poorer overall survival and disease-free survival (DFS) rate than patients without MCM8 alterations (Figure 5D, 5E). Using the muTarget database, mutation status of TP53, ABCB5, CUBN and RB1 were determined to correlate with dysregulation of MCM8 mRNA expression in HCC patients (Figure 5F).

### Lower DNA methylation status correlated with upregulation of MCM8 expression and poor survival in HCC patients

**Figure 6:**
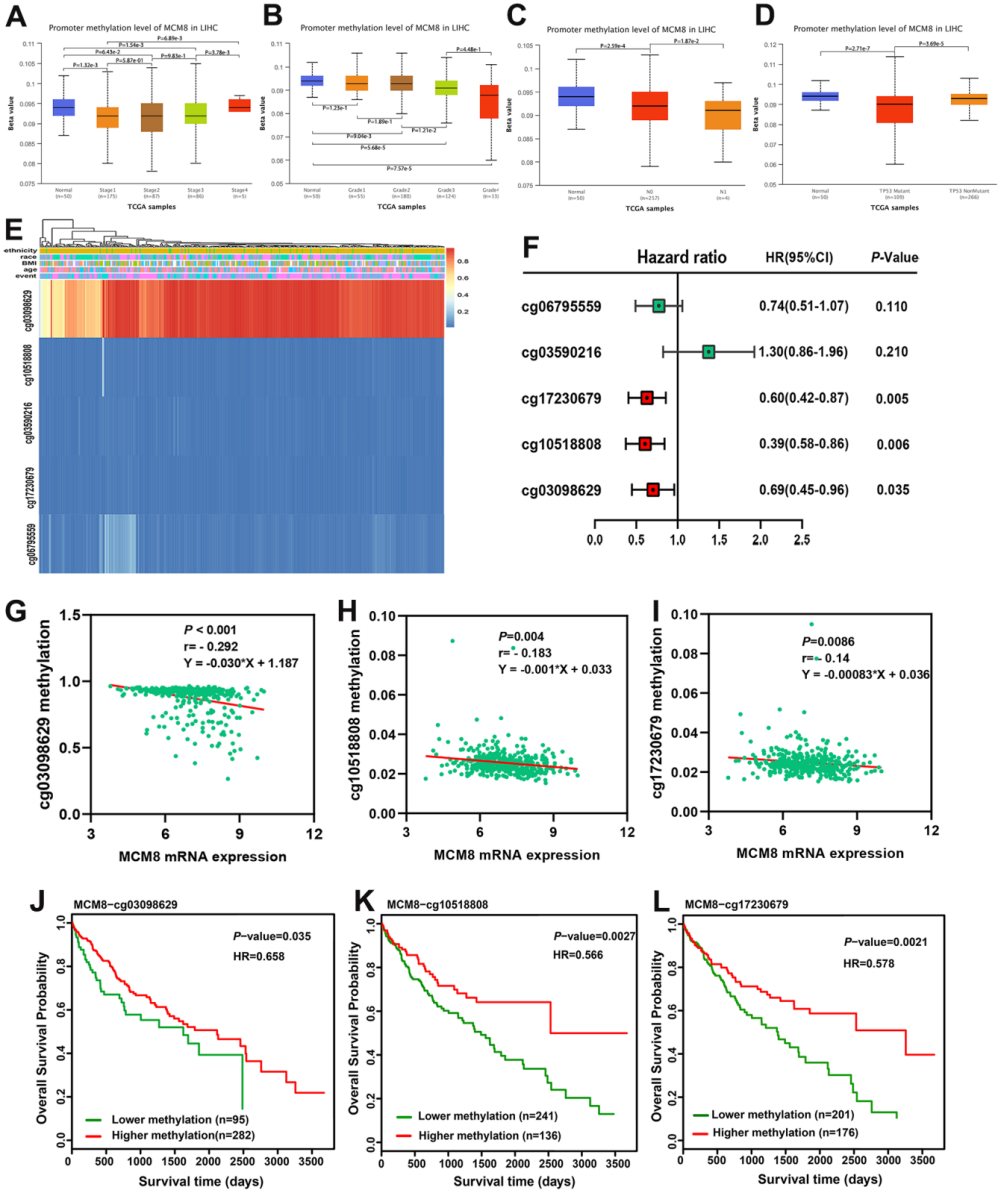
**Hypomethylation status correlated with upregulation of MCM8 expression and poor survival in HCC patients.** (**A**, **B**) DNA methylation level of MCM8 gradually decreased with the progression of tumor stages (**A**) and histologic grades (**B**). (**C**) The MCM8 methylation level is lower in metastatic lymph nodes than without metastasis. (**D**) The MCM8 methylation level is lower in HCC with TP53 mutation. (**E**) The heat map shows CCT7-related methylated CpG sites in HCC. (**F**) Methylation level of three CpG sites were associated with overall survival times of HCC patients. (**G**–**I**) MCM8 mRNA expression negatively correlated with methylation levels of cg03098629 (**G**), cg10518808 (**H**) and cg17230679 (**I**). (**J**–**L**) Hypermethylation of cg03098629 (**J**), cg10518808 (**K**) and cg17230679 (**L**) was associated with better overall survival times.

DNA methylation of MCM8 in HCC tissues was lower than in adjacent normal liver tissues, and further analyses revealed that DNA methylation of MCM8 gradually decreased with the tumor stages (Figure 6A) and histologic grades (Figure 6B) increased. In metastatic lymph nodes, the MCM8 methylation level still decreased (Figure 6C). It is interesting to note that there was a significant correlation between the MCM8 methylation level with TP53 mutation (Figure 6D). Using the MethSurv database, five MCM8-related methylation CpG sites were identified in HCC, including cg06795559, cg03590216, cg17230679, cg10518808 and cg03098629 (Figure 6E). The univariate Cox regression analysis demonstrated that three methylation CpG sites were risk factors for the overall survival times of HCC patients (Figure 6F). In addition, the correlation analysis demonstrated that the methylation status of these three CpG sites were negatively correlated with MCM8 mRNA expression (Figure 6G–6I). Furthermore, as shown in the survival curves, HCC patients with hypermethylation status of these three CpG sites have better overall survival than patients with hypomethylation status (Figure 6J–6L).

### Identification of MCM8 co-expressed genes in HCC

**Figure 7:**
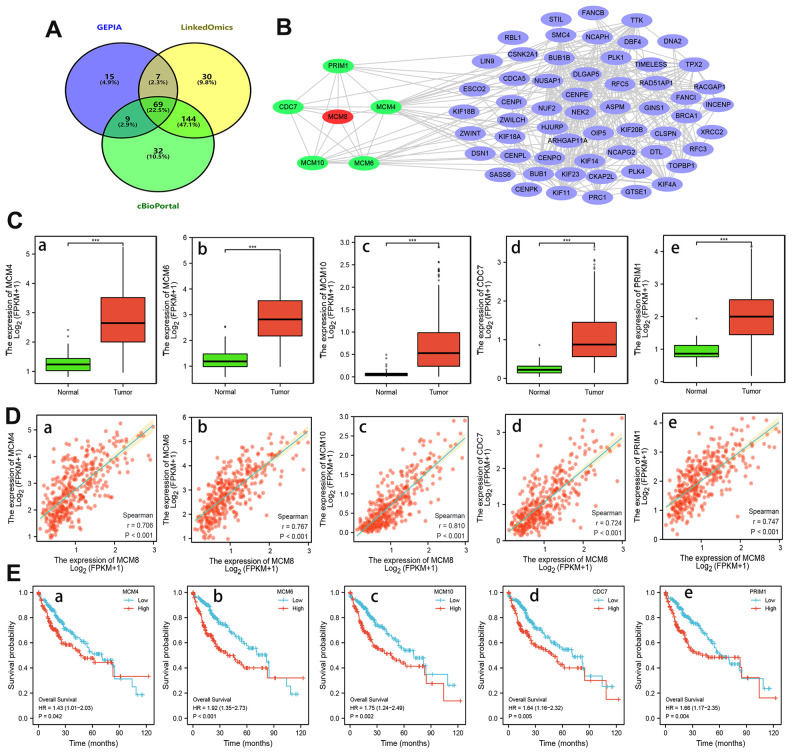
**Analysis of MCM8 co-expressed genes in HCC.** (**A**) 69 overlapping co-expressed genes of MCM8 were identified from the GEPIA, LinkedOmics and cBioPortal database. (**B**) The PPI network showed that the MCM4, MCM6, MCM10, CDC7 and PRIM1 protein directly interacted with MCM8. (**C**) MCM4 (**a**), MCM6 (**b**), MCM10 (**c**), CDC7 (**d**) and PRIM1 (**e**) mRNA were overexpressed in HCC. (**D**) Correlation of MCM8 mRNA levels with MCM4 (**a**), MCM6 (**b**), MCM10 (**c**), CDC7 (**d**) and PRIM1 (**e**) mRNA levels. (**E**) Higher MCM4 (**a**), MCM6 (**b**), MCM10 (**c**), CDC7 (**d**) and PRIM1 (**e**) mRNA expression predicted poorer overall survival times of HCC patients. ***P<0.001.

We identified the genes that positively correlated with MCM8 mRNA expression in the GEPIA, LinkedOmics and cBioPortal, respectively. A total of 69 overlapping genes from the three databases with Spearman’s values greater than 0.70 were determined as co-expressed genes of MCM8 (Figure 7A). Using the STRING database and Cytoscape software, a PPI network with 64 nodes and 314 edges was constructed. We noticed that five proteins (MCM4, MCM6, MCM10, cell division cycle 7 (CDC7) and primase-DNA-polypeptide 1 (PRIM1)) directly interacted with MCM8 in the PPI network (Figure 7B). Next, TCGA database was applied to investigate the mRNA expression of these co-expressed genes and found its expression significantly higher in HCC than in normal liver tissues (Figure 7C). Subsequently, we investigated the correlation between these five genes and MCM8 expression using TCGA and validated the remarkably positive correlations (Figure 7D). In addition, the Kaplan–Meier curves suggested that higher mRNA expression of genes was significantly associated with shorter overall survival times in patients with HCC (Figure 7E).

### Elevated expression of MCM8 was regulated the cell cycle and DNA replication signaling in HCC

**Figure 8:**
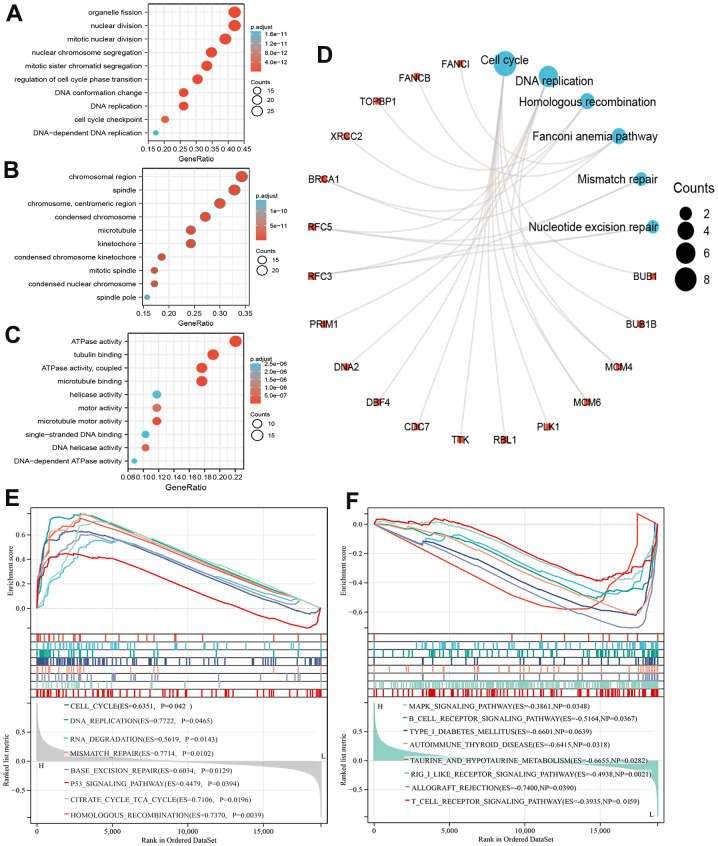
**Functional enrichment analysis of MCM8 in HCC.** (**A**–**C**) The bubble diagram for the biological process (**A**), cellular component (**B**), and molecular function (**C**) items in GO analysis on co-expression genes of MCM8. (**D**) Results of KEGG enrichment analysis on co-expression genes of MCM8. (**E**, **F**) GSEA results of significant signaling pathways that high (**E**) and low (**F**) MCM8 expression data sets enriched.

We performed the GO and KEGG enrichment analysis on 69 co-expressed genes using the DAVID web server to explore the mechanism and signaling pathway whereby MCM8 involve in the tumorigenesis and progression in HCC. In the GO Biological Process items, these genes were related mainly to process of mitotic and cell cycle, such as organelle fission, mitotic nuclear division, regulation of cell cycle phase transition, DNA replication, and cell cycle checkpoint, etc. (Figure 8A). In the GO cellular component items, these genes were mainly involved in chromosomal region, mitotic spindle, microtubule, condensed nuclear chromosome, spindle pole, etc. (Figure 8B). For the GO molecular function items, these genes were mainly related to ATPase activity, tubulin binding, microtubule-binding, helicase activity, motor activity, DNA helicase activity, etc. (Figure 8C). In addition, KEGG revealed that the most significant pathways of MCM8 co-expressed genes enriched were related mainly to the Cell cycle and DNA replication. Other pathways included Homologous recombination, Fanconi anemia pathway, Mismatch repair, and Nucleotide excision repair (Figure 8D).

We also performed GSEA to identify signaling pathways related to MCM8 expression. RNAseq data were downloaded from TCGA database and stratified into high and low MCM8 expression data sets. Results showed that high MCM8 expression data sets enriched mainly in Cell cycle, DNA replication, RNA degradation, Mismatch repair, Base excision repair, P53 signaling pathway, etc. (Figure 8E). Meanwhile, the low MCM8 expression data sets enriched mainly in MAPK signaling pathway, B cell receptor signaling pathway, Autoimmune thyroid disease, T cell receptor signaling pathway, etc. (Figure 8F). We next performed GSEA analyses in the LinkedOmics web server to illustrate the biological processes related to MCM8. We displayed the top 50 hallmark gene sets in Supplementary Figure 1A. In addition, the “DNA replication”, “chromosome segregation”, and “cell cycle” signaling pathways were significantly enriched, with normalized enrichment scores (NESs) of 2.092, 2.058, and 1.874, all normalized p values (NOM p values) lower than 0.001 (Supplementary Figure 1B–1D).

### Correlation analysis between dysregulation of MCM8 expression and tumor-infiltrating immune cells in HCC

**Figure 9:**
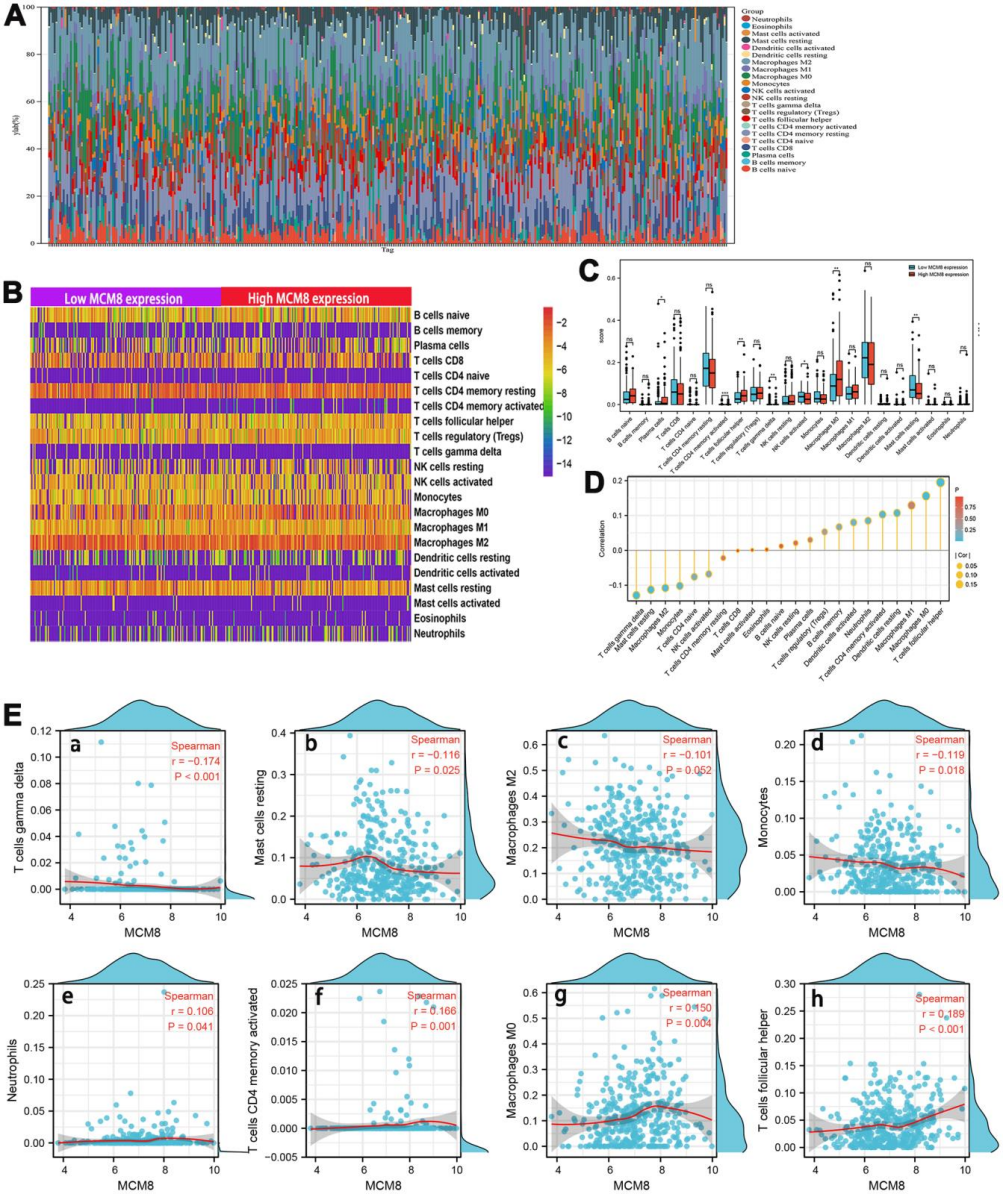
**Association between MCM8 mRNA expression and immune infiltration in HCC.** (**A**) Estimated fractions of 22 immune cells in each HCC tissue, where different color represented different cell types. (**B**) The heat map shows the difference in immune cells infiltration between high and low SNRPA expression HCC samples. (**C**) The comparison of estimated fractions of 22 immune cells between the high and low MCM8 expression samples. (**D**) Correlation between MCM8 expression and 22 tumor-infiltrating immune cells in HCC using the ssGSEA algorithm. (**E**) The correlation of MCM8 expression with immune infiltration level of T cells gamma delta (**a**), Mast cells resting (**b**), Macrophages M2 (**c**) and Monocytes (**d**), Neutrophils (**e**), T cells CD4 memory activated (**f1**), Macrophage M0 (**g**), and T cells follicular helper (**h**). *P<0.05, **P<0.001, ***P<0.001, ns: no statistically significant.

A growing body of evidence suggested that the composition of the tumor microenvironment and immunocyte infiltration ratio plays an essential role in tumorigenesis and progression in patients with HCC [29–31]. As such, we explored the correlation between MCM8 expression and 22 tumor-infiltrating immune cells using the CIBERSORT algorithm and ssGSEA. Estimated fractions of 22 immune cells in each HCC tissue was calculated and visualized in a bar chart, and different color represented different cell types (Figure 9A). In addition, the difference in immune cells infiltration between low and high MCM8 expression samples was investigated and exhibited in a heat map (Figure 9B). Using the CIBERSORT algorithm, high MCM8 expression samples have a higher proportion of plasma cells, T cells CD4 memory activated, T cells follicular helper, Macrophages M0, whereas a lower proportion of T cells gamma delta, NK cells activated, and Mast cells resting (Figure 9C).

Next, the correlation between MCM8 expression and 22 tumor-infiltrating immune cells in HCC was also investigated by employing the ssGSEA algorithm with Spearman’s analysis (Figure 9D). Results suggested that MCM8 expression may be negatively associated with infiltration levels of T cells gamma delta (r=-0.174, P<0.001), Mast cells resting (r=-0.116, P=0.025), Macrophages M2(r=-0.102, P=0.052) and Monocytes (r=-0.119, P=0.018) (Figure 9E a–d). Whereas, MCM8 expression may be positively associated with infiltration levels of Neutrophils (r=106, P=0.041), T cells CD4 memory activated (r=0.166, P=0.001), Macrophage M0 (r=0.150, P=0.004), and T cells follicular helper (r=0.189, P<0.001) (Figure 9E e–h). All results suggested that MCM8 potential regulates the extent of immune cell infiltration in HCC. We believe that there is a need for further experimental and theoretical studies in order to validate the associations between MCM8 expression with immune cell infiltration.

### MCM8 expression at the single-cell level

**Figure 10:**
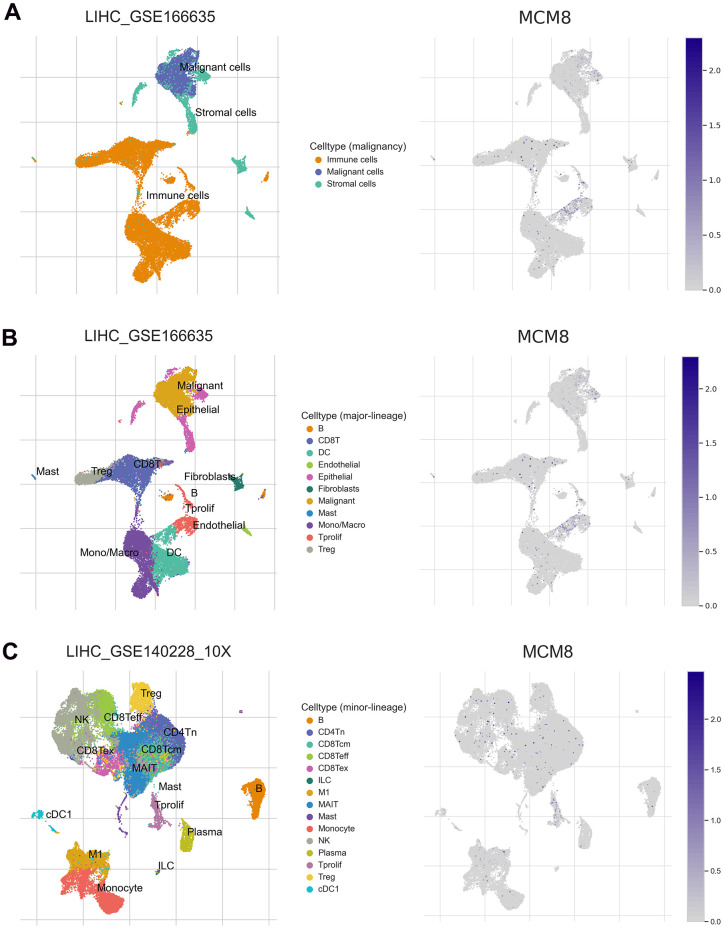
MCM8 expression at the single-cell level (**A**) MCM8 was detected at a high level in malignant and immune cells in the LIHC_GSE166635 dataset. (**B**) Endothelial and DC cells have high levels of MCM8 mRNA in the LIHC_GSE166635 dataset. (**C**) MCM8 was mainly detected in the Tprolif, monocytes, and macrocytes in the LIHC_GSE140228_10X dataset.

We next investigated the MCM8 mRNA level using single-cell RNA sequencing analysis. In the LIHC_GSE166635 dataset, MCM8 was detected at a high level in malignant and immune cells (Figure 10A). In addition, the LIHC_GSE166635 dataset suggested that endothelial and DC cells have high levels of MCM8 mRNA (Figure 10B). In the LIHC_GSE140228 dataset, MCM8 was mainly detected in the Tprolif, monocytes, and macrocytes (Figure 10C). These results revealed that there are interactions between immune dysfunctions with MCM8 dysregulation.

## DISCUSSION

MCM8, one of the critical proteins of DNA-replication-licensing factors of the MCM family, is responsible for DNA-replication initiation and elongation [32]. In add MCM8 was reported associated with chromosomal instability [17]. Lin et al. demonstrated that MCM8 was negatively regulated by CREB-miR-630 and plays a critical role in the DNA homologous recombination repair in human liver cells [33]. MCM8 mRNA was found expressed in the placenta, lung, and liver, and its expression is altered in certain forms of neoplasia [34]. Some studies point out that cancer cells are subjected to greater stress of DNA replication and mitotic than normal cells due to the high growth stimulus of carcinogenesis, and that MCMs play a critical role in the repair process of DNA replication and mitotic stress [35, 36]. Indeed, amplifications and mutations of the MCMs genes lead to genomic structural alterations, which could result in tumorigenesis and progression of multiple cancer types [37]. In addition, MCM8 has been found to be copy number amplified and overexpressed in various human malignancies, and increased MCM8 correlated with aggressive characteristics of these malignancies [38]. Zhu et al. reported that MCM8 overexpressed in bladder cancer and promoted proliferation and metastasis by regulating AKT/MAPK9 signaling pathway [27]. Existing study suggested that MCM8 was regulated by EGFR signaling and interacted with DNA-replication-initiating factors to promote the growth of glioma stem cells [25]. However, the role and the mechanism of action of MCM8 in the tumorigenesis and progression of HCC remains far from being fully elucidated.

Using data from public databases and a cohort of 132 HCC patients, both MCM8 mRNA and protein were significantly overexpressed in HCC tissues than in adjacent normal liver tissues. In addition, correlation analysis suggested that high MCM8 expression correlated with poor clinicopathologic characteristics. More importantly, both the upregulation of MCM8 mRNA and protein were valuable independent prognostic indicators in patients with HCC. Survival analysis also indicated that higher MCM8 expression correlated with shorter OS and RFS. These results suggested that MCM8 may be a prognostic biomarker in HCC. Therefore, postoperative MCM8 IHC detection may be an effective method to predict the prognosis after curative resection, but its clinical utility requires further validation.

Growing evidence points out that the dysregulation of gene expression was extensively caused by genetic alteration and aberrant DNA methylation levels [39–41]. Herein, we investigated the genetic alteration and DNA methylation of MCM8 in HCC. Using the cBioPortal database, 60% of queried HCC patients have genetic alterations in MCM8, and a mutation hotspot of H161Q/Missense was identified. In addition, such alterations predicted a poorer OS and DFS. We also identified three CpG sites (cg17230679, cg10518808 and cg03098629), and that its hypomethylation correlated with upregulation of MCM8 expression and poor OS. Thus, it was reasonable to speculate that MCM8 alteration and DNA methylation cause the dysregulation of MCM8 expression. Currently, targeting DNA damage repair pathways caused by DNA replication mutations has been an effective therapeutic target for cancer treatment [42, 43]. Therefore, drugs designed to address alterations of MCM8 may be an effective strategy for the treatment of HCC. We then established a PPI network and identified five co-expressed genes of MCM8 including CDC7, PRIM1, MCM4, MCM6, and MCM10, which directly interacted with MCM8. It is widely accepted that genes with similar expression patterns may be functionally similar. The survival curves demonstrated that higher expression of these co-expressed genes correlated with shorter OS periods in HCC patients. These findings suggested that MCM8 may as an oncogene in the process of HCC tumorigenesis and development.

We then explored the mechanism and signaling pathway whereby MCM8 involving in the tumorigenesis and progression in HCC. Both KEGG and GSEA demonstrated that MCM8 participated in DNA replication and cell cycle signaling pathways. DNA replication signaling has already been shown to contribute remarkably to the tumorigenesis and progression of multiple cancer types, and our results were consistent with previous studies in lung adenocarcinoma and myeloid tumors [28, 44]. Existing studies suggested that MCM6 and MCM10 promoted the progression by regulating cell cycle signaling [12, 15]. From our results, MCM6 and MCM10 were co-expressed genes of MCM8, and it is reasonable to speculate that MCM8 may be a cell-cycle regulator in HCC. More importantly, KEGG and GSEA preliminarily validated this hypothesis and further experimental validation was required.

This study also investigated the correlation between MCM8 expression and infiltration status of 22 immune cells in HCC using the CIBERSORT algorithm and ssGSEA. The CIBERSORT algorithm demonstrated that MCM8 expression positively correlated with infiltration of Neutrophils, T cells CD4 memory activated, Macrophage M0 and T cells follicular helper, whereas negatively correlated with infiltration of T cells gamma delta, Mast cells resting, Macrophages M2 and Monocytes. Zhou et al. reported that tumor-associated neutrophils promoted the proliferation and progression of HCC through recruiting tumor-associated neutrophils [45]. In addition, higher infiltration status of T cells follicular helper is associated with proliferation of HCC and promoted the progression [46]. A recent paper published in Nature communication found that higher intratumoural frequencies of T cells gamma delta was associated with enhanced HCC patient survival [47]. This suggested that high MCM8 expression plays a key role in dysregulation of these immune cells, and therefore influences the prognosis of patients with HCC. However, we believe that there is a need for further experimental and theoretical studies in order to validate the associations between MCM8 expression with immune cell infiltration.

In conclusion, our study demonstrated that MCM8 mRNA and protein were significantly highly expressed in HCC tissues and MCM8 could be an independent prognostic biomarker for clinical outcomes in HCC patients. The upregulation of MCM8 may be correlated with the hypomethylation of the CpG site cg17230679, cg10518808 and cg03098629. In addition, MCM8 was an oncogene that promotes HCC tumorigenesis and progression through the DNA replication and cell cycle signaling pathways. High MCM8 expression may play a substantial role in dysregulation of infiltration status of immune cells, and therefore influences the prognosis of patients with HCC, but further experimental and theoretical studies were required.

## MATERIALS AND METHODS

### Profiling of MCM8 mRNA expression in various public databases

We investigated the MCM8 mRNA expression in HCC and adjacent normal livers tissues using datasets from TCGA [48] and GEO (GSE54236 and GSE76427 datasets) databases [49]. The receiver operating characteristic (ROC) curves were plotted to evaluate the diagnostic significance of MCM8 in distinguishing between HCC and normal liver tissues. In addition, we investigated the correlation between MCM8 mRNA expression with the OS, RFS, progression-free survival (PFS), and disease-specific survival (DSS) in the Kaplan Meier plotter database, which capable to evaluate the effect of 54k genes (mRNA, miRNA, protein) on survival in 21 cancer types [50]. Furthermore, we used the Cox regression analysis to determine the prognostic value of the MCM8 mRNA level.

### Prognostic significance analysis of MCM8 protein expression

We performed immunohistochemical staining assay to investigate the prognostic significance of MCM8 protein expression in HCC. We collected 132 HCC specimens and relevant complete clinicopathologic characteristics from the patients who underwent curative liver resection from January 2013 to December 2015 at Taizhou Central Hospital. All HCC specimens were stored by formalin-fixed paraffin-embedded blocks. The complete clinicopathologic characteristics, including basic clinical features (age, gender, serum α-fetoprotein level, tumor treatment history, etc.) and the following information (tumor number, tumor lesion size, Tumor grade, histopathological differentiation, etc.), were obtained from the electronic medical record (EMR) systems of the hospital. Survival data were acquired through repeat admissions, telephone follow-up, and the Social Security Death Index. All patients met our inclusion criteria as follows: only one cancer lesion or multiple lesions but limited to one hepatic lobe, Child-Pugh class A or B, without any history of cancer treatment prior to hepatectomy, the pathological diagnosis of HCC. All sampling was done in accordance with the relevant medical ethics regulations and approved by the ethics committee of Taizhou Central Hospital (Taizhou, China). In addition, we obtained written informed consent from all participants prior to surgery.

### Immunohistochemistry (IHC) assay and evaluation

The 132 formalin-fixed paraffin-embedded blocks were cut into 4 -μm sections and rehydrated and deparaffinized by malondialdehyde and ethanol. Then, the sections were immersed in a boiled 0.01 M citrate buffer (pH 6.0) for 20 min to perform the antigen retrieval. Next, the sections were immersed in a 3% H2O2 for 10 min to block endogenous peroxidase activity. The sections were incubated with the anti-human MCM8 polyclonal antibody (PA5-41325; 1:300; Thermo Fisher Scientific, USA) at 4° C overnight and then incubated with a secondary antibody (1:50,000; KIT-5010; anti-rabbit/mouse IgG; Maixin Biotechnology Development Co., Ltd., China) at room temperature for 25 min. The 3,3’-diaminobenzidine (DAB) and hematoxylin were used to stain and counterstain sections. Brownish-yellow granules in the cytoplasm and/or nucleus represented positive staining. The sections only incubated with secondary antibodies without primary antibodies were set as the negative control. All IHC staining was assessed by two separate experienced pathologists who do not know in advance any information on the patient’s clinical condition and diagnosis. Evaluation of the IHC staining based on a semi-quantitative IHC scoring system with 0-4 points scale: 0, no positive cells; 1, 0-25% positive cells, 2, 26-50% positive cells; 3, 51-75% positive cells; and 4, >75 positive cells. HCC samples with a IHC score of 0, 1 or 2 was defined as low MCM8 expression group, whereas a score of 3 or 4 was defined as high MCM8 expression group.

### Establishment and validation of the predictive nomogram

All Independent risk factors of OS and RFS identified by the multivariate Cox regression analysis were integrated to establish a nomogram. Next, we plotted the calibration curves of 1-, 3-, and 5-year OS and RFS probability to discriminate the predicted probabilities and actual probabilities. Then, the decision curve analysis (DCA) based on a nomogram was performed to help make decisions in clinical practice for the acquisition of the best net benefit.

### Genetic alteration of MCM8 in HCC

We investigated the copy number variation and mutation of MCM8 using the Liver Hepatocellular Carcinoma (TCGA, Firehose Legacy) dataset downloaded from the cBioPortal, an open access repository of cancer genomics datasets [51, 52]. Then, we investigated the effect of genetic alteration on gene expression of MCM8 and prognosis in patients with HCC. The muTarget, a database that connects mutation status to gene expression changes in solid tumors, was employed to identify the genes mutation associated with dysregulation of MCM8 expression [53].

### DNA methylation of MCM8 in HCC

The UCLCAN database was employed to analyze the association between DNA methylation of MCM8 with mRNA expression in different stages and grades [54]. In addition, the MethSurv database was used to identify the CpG sites that correlated with mRNA expression and overall survival probability [55]. The Spearman correlation analysis was applied to determine the correlation between the methylation status of CpG sites and MCM8 expression. The methylated data was downloaded from Illumina Human Methylation 450 datasets (https://xenabrowser.net/datapages/) in TCGA database.

### Co-expressed genes identification of MCM8

We screened out the genes that positively correlated with MCM8 mRNA expression in the GEPIA, LinkedOmics and cBioPortal, respectively [56, 57]. Then, the overlapping genes with the Spearman’s correlation value greater than 0.7 from three databases were determined as co-expressed genes of MCM8. Then, these co-expressed genes were input into the STRING database to investigate the interaction between proteins and proteins [58]. We next visualized a protein-protein interaction (PPI) network in the Cytoscape software (Version 3.9.1). In addition, the genes that directly interacted with MCM8 were selected and used for the next steps in our research.

### Functional enrichment analysis of GO, KEGG and GSEA

In order to explore the gene functions and potential signaling pathways in tumorigenesis and progression of HCC, we performed GO and KEGG enrichment analysis using the Functional Annotation Tool in DAVID database on co-expressed genes of MCM8, which were identified from three databases. Transcription profiling mRNA data from 374 HCC samples were obtained from TCGA to perform GSEA enrichment analysis. We stratified the HCC patients into high MCM8 expression group and low MCM8 expression group taking the cutoff value of the median MCM8 mRNA expression value. During execution process, “c2.cp.kegg.v7.0.symbols.gmt” was selected as the functional gene set, and the number of permutations was set to 1000. When P<0.05 and a false discovery rate <0.25, the critical value of the significant gene functions and pathways were considered statistically significant.

### Single-cell RNA sequencing

We employed the TISCH2 web server to conduct single-cell RNA sequencing analysis and investigate expressed differences of MCM8 mRNA in malignant cells and different types of immune cells. Two datasets (LIHC_GSE166635, LIHC_GSE1402228) were selected to display the interactions between immune dysfunctions with MCM8 dysregulation.

### Immune infiltration analysis

We performed the immune infiltration analysis using transcription profiling mRNA data from 374 HCC samples in TCGA database. We calculate the proportion of 22 types of immune cells in each HCC sample using the CIBERSORT software. In addition, the single sample GSEA (ssGSEA) method with the “GSVA” R package was applied to analyze the level of tumor immune infiltration. Then, we investigated the association between the level of MCM8 mRNA expression and the abundance of 22 infiltrating immune cell types.

### Statistical analysis

R (v.3.6.3) software was employed to perform statistical analysis and plot figures. Pearson’s chi-square test, two-tailed Student’s t-tests, or Wilcoxon test were applied to analyze the correlation between MCM8 expression level and clinicopathological characteristics. The univariate and multivariate Cox regression analysis was employed to determine the prognostic value of MCM8. Correlations analyses were performed using Spearman correlation tests. The Kaplan–Meier method with log-rank test was used for comparison of survival rate. P < 0.05 was considered statistically significant.

### Availability of data and materials

The datasets generated for this study can be found in the GEO database (https://www.ncbi.nlm.nih.gov/geo/) and TCGA database (https://portal.gdc.cancer.gov).

## Supplementary Material

Supplementary Figure 1
