# Supplementary material for: High MCM8 expression correlates with unfavorable prognosis and induces immune cell infiltration in hepatocellular carcinoma
Source: Aging (Albany NY). 2022 Dec 27;14(24):10027–49. doi: 10.18632/aging.204440 (PMC9831725; doi:10.18632/aging.204440)
Supplement: Supplementary Figure 1 [file aging-14-204440-s001.pdf]

SUPPLEMENTARY FIGURE

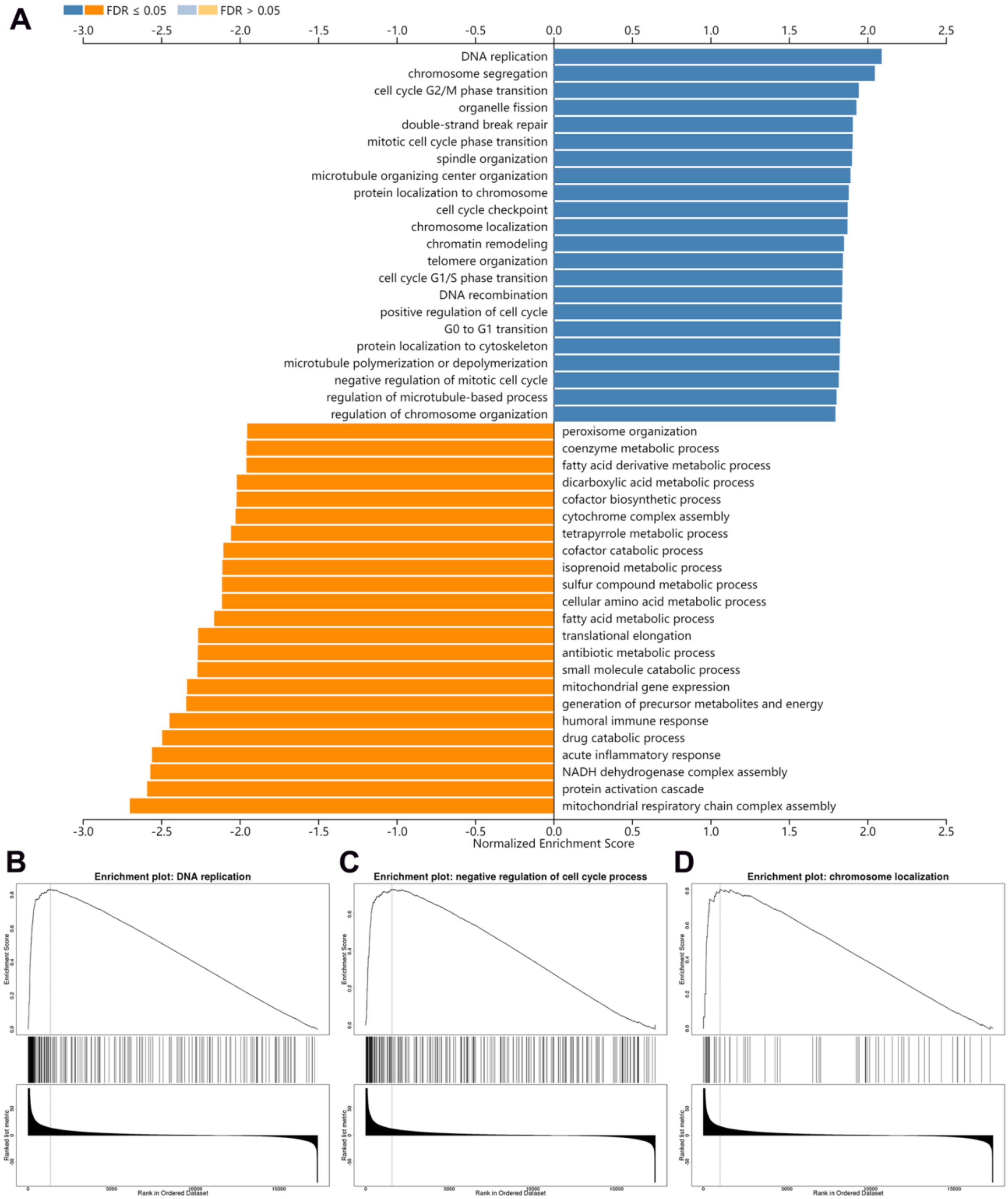

Supplementary Figure 1. Gene set enrichment analyses (GSEA) and gene set variation analyses (GSVA) to illustrate the biological processes related to MCM8. (**A**) Barplot of GSVA results of top 50 biological processes that MCM8 related. (**B**–**D**) GSEA results of the "DNA replication", "chromosome segregation", and "cell cycle".
